# Supplementary material for: Dopamine signaling enriched striatal gene set predicts striatal dopamine synthesis and physiological activity in vivo
Source: Nat Commun. 2024 Apr 30;15:3342. doi: 10.1038/s41467-024-47456-5 (PMC11061310; doi:10.1038/s41467-024-47456-5)
Supplement: Supplementary file 1 — Supplementary Information [file 41467_2024_47456_MOESM1_ESM.pdf]

Supplementary Information for:

Dopamine signaling enriched striatal gene set predicts striatal dopamine synthesis and physiological activity in vivo

Authors:

Leonardo Sportelli[1,2,#], Daniel P. Eisenberg[3, #], Roberta Passiatore[2], Enrico D'Ambrosio[2,4], Linda A. Antonucci[2], Jasmine S. Bettina[3], Qiang Chen[1], Aaron L. Goldman[1], Michael D. Gregory[3], Kira Griffiths[4,5], Thomas M. Hyde[1,6,7], Joel E. Kleinman[1,7], Antonio F. Pardiñas[8], Madhur Parihar[1], Teresa Popolizio[9], Antonio Rampino[2,10], Joo Heon Shin[1], Mattia Veronese[11,12], William S. Ulrich[1], Caroline F. Zink[13], Alessandro Bertolino[2,10], Oliver D. Howes[4], Karen F. Berman[3], Daniel R. Weinberger [1,6,7,14,15,*], Giulio Pergola [1,2,7,*]

Affiliations:

[1] Lieber Institute for Brain Development, Johns Hopkins Medical Campus, Baltimore, MD, USA.

[2] Group of Psychiatric Neuroscience, Department of Translational Biomedicine and Neuroscience, University of Bari Aldo Moro, Bari, Italy.

[3] Clinical and Translational Neuroscience Branch, National Institute of Mental Health, Intramural Research Program, NIH, DHHS, Bethesda, MD, USA.

[4] Department of Psychosis Studies, Institute of Psychiatry, Psychology and Neuroscience, King's College London, London, SE5 8AF, UK.

[5] Holmusk Technologies, New York, NY, USA.

[6] Department of Neurology, Johns Hopkins University School of Medicine, Baltimore, MD, USA.

[7] Department of Psychiatry and Behavioral Sciences, Johns Hopkins University School of Medicine, Baltimore, MD, USA.

[8] MRC Centre for Neuropsychiatric Genetics and Genomics, Cardiff University, Cardiff, UK.

[9] Radiology Department, IRCCS Ospedale Casa Sollievo della Sofferenza, Italy.

[10] Azienda Ospedaliero Universitaria Consorziale Policlinico, Bari, Italy.
[11] Department of Information Engineering, University of Padua, Italy.
[12] Department of Neuroimaging, Institute of Psychiatry, Psychology and Neuroscience, King's College London, London, SE5 8AF, UK.
[13] Baltimore Research and Education Foundation, Baltimore, MD, USA.
[14] Department of Neuroscience, Johns Hopkins University School of Medicine, Baltimore, MD, USA.
[15] Department of Genetic Medicine, Johns Hopkins University School of Medicine, Baltimore, MD, USA.

# These authors contributed equally: Leonardo Sportelli, Daniel P. Eisenberg

*Corresponding authors
Giulio Pergola, PhD
855 North Wolfe St
21205 Baltimore, MD
Giulio.Pergola@libd.org
Giulio.Pergola@uniba.it

Please address correspondence to:
Daniel R. Weinberger, MD
855 North Wolfe St
21205 Baltimore, MD
Daniel.Weinberger@libd.org

This File includes:

Supplementary Methods, Supplementary Figures 1-8, Supplementary Table 1, Supplementary References.

Supplementary Methods

Genotype data processing

LIBD, NIH and UNIBA cohorts. Participants underwent blood withdrawal for subsequent DNA extraction from peripheral blood mononuclear cells. To this aim, approximately 20ml of fresh blood was obtained through a conventional venous blood collection with 10ml EDTA Vacutainer Venous Blood Collection Glass Tubes (Vacutainer ®). Approximately 200 ng DNA was used for genotyping analysis. DNA was concentrated at 50ng/µl (diluted in 10 mM Tris/1mM EDTA) with a Nanodrop Spectrophotometer (ND-1000). Samples were genotyped using variate Illumina Bead Chips including 510K/610K/660K/2.5M.

Quality control was performed on the cohorts separately using PLINK (version 2; http://pngu.mgh.harvard.edu/purcell/plink/)[1] according to standards developed by the Psychiatric Genomics Consortium[2] including SNP missingness < 0.05 (before sample removal); subject missingness < 0.02; autosomal heterozygosity deviation (|Fhet| < 0.2; for NIH cohort |Fhet| within 3.5 standard deviations); SNP missingness < 0.02 (after sample removal), SNP Hardy-Weinberg equilibrium (HWE: P > 10−6) and minor allele frequency (MAF) > 0.01. Furthermore, the degree of recent shared ancestry, i.e., the identity by descendent (IDB)[3], has been estimated within the cohorts to define the relatedness of all pairs on individuals through the PLINK function '--genome'[4]. The threshold 0.125 (for NIH cohort 0.185) represents the relatedness of 3rd degree[5] that was used as cut-off to exclude possible influence of relatedness within cohorts on dependency between observations[6]. Of each pair of related individuals, the one belonging to the group with greater numerosity within each cohort is dropped from the final datasets.

Genotype imputation was performed using the pre-phasing/imputation stepwise approach implemented in IMPUTE2 / SHAPEIT (default parameters and chunk size of 3 Mb for LIBD and UNIBA - 250 Kbp for NIH) and using Phase 3 1000 genome as reference panel[7, 8]. After imputation, imputed dosage data for each SNP with imputation quality (INFO) > 0.9 were used for polygenic risk scores (PRS) calculation.

For LIBD cohort, the first 10 principal components of the whole genome data were calculated using EIGENSOFT v5.01 (EIGENSOFT, http://www.hsph.harvard.edu/alkes-price/software/) and considered as nuisance covariates in genetic analysis while for UNIBA and NIH cohorts the *SNPRelate* R package[9] and PLINK (version 2) were respectively used..

KCL cohort. DNA was extracted from whole blood samples or cheek swabs using standard procedures[10]. Genotyping was performed at Cardiff University, using HumanCore Exome 1.1 arrays ("Psych-chip", Illumina, San Diego, California, USA). Genotype quality control (QC) was performed according to standard parameters[11].

PLINK (version 1.9) was used to implement pre-imputation quality control procedures. Individuals were excluded from the sample if estimates of the inbreeding coefficient F indicated ambiguous sex (F = 0.2 - 0.8), or if there was a discrepancy between their genotypic and self-reported sex. Any discrepancies were checked with the data collection site to confirm no errors were made during manual entry of phenotypic data. Samples with a genotype call < 95%, or high pairwise relatedness (pi-hat > 0.1) were excluded. SNPs were removed if MAF < 0.01, if deviation from HWE at p-mid ≤ 1e-6, or if minimum marker call rate < 95%.

Chromosome files were uploaded to the Michigan Imputation Server (https://imputationserver.sph.umich.edu/index.html#!) where they underwent in house quality control before being passed through imputation pipeline (see: https://imputationserver.readthedocs.io/en/latest/). Data passing QC were phased using Eagle[12] v2.4 and imputed using minimac4 (https://genome.sph.umich.edu/wiki/Minimac4) with the HRC-r1.1 reference panel[13] (GRCh37/hg19 array build), with population set to mixed. SNPs were excluded if the imputation INFO score was < 0.9, MAF < 0.10, or HWE P < 1e-6, genome = 0.05.

The Principal Components Analysis in Related samples (PC-AiR) method[14] was used in R (GENESIS R/Bioconductor package[15]) on the full set of genotypes to generate the top 10 principal components of the sample.

SDA run and post-processing

We run SDA 10 times using the following parameters:

-- N = 238 (number of samples)

-- max_iter = 3000 (number of iterations)

-- num_comps = 238 (number of components)

-- seed = 400 (used for results replication)

Running the method multiple times, we obtained some components that are found consistently across multiple runs, whereas other components only occur in a small number of them. To identify robust components, the authors of the paper implemented a method that clusters similar components across different runs. We then focused on large clusters containing components from multiple different runs and used these as the basis for further analyses. More specifically, at the end of each run we stored the individual and tissue scores, gene loadings and Posterior Inclusion Probabilities (PIPs = a ranking measure to assess whether the data favors the inclusion of a variable in the regression model, i.e., the probability that, after the SDA decomposition, each gene with his loading or weight belongs to a specific component). After the 10 runs finished, following what the authors did in the paper, we calculated the absolute correlation between the individual scores for all pairs of components across the ten runs. Hierarchical clustering was then used to group components into clusters, using one minus the absolute correlation as a dissimilarity measure. The clustering was terminated when no correlations between clusters were above 0.4. Although authors of SDA used 0.6 as threshold, we decided to use a more lenient one to maximise the information considering our significantly smaller sample size. The components within each cluster were then combined. We took the mean of the individual scores, tissue scores and gene loadings and the median PIPs to obtain the most representative value within each cluster (centroid). We finally obtained 126 robust components and we used PIP threshold > .5 to identify genes within each component.

Parsed-PRS association with KCL PET data across different ancestry clusters

Since the KCL discovery cohort was the most heterogeneous in terms of ethnicities included (Supplementary Table 1), together with the ancestry stratification reported in the main text for the main analyses, we also decided to evaluate different ancestry subdivisions based on the visualization of the first two PCA dimensions, i.e. top axes of variation. Following what we reported in the main text (see Methods), we used a procedure developed by the ENIGMA consortium that consists in performing a PCA on target data merged with the HapMap121 phase 3 reference dataset (https://enigma.ini.usc.edu/wp-content/uploads/2012/07/ENIGMA2_1KGP_cookbook_v3.pdf). For this analysis we included all KCL samples whose genotype information was available (N: 168). We then plotted the first two PCs against each other and defined two different clusters: one excluding individuals belonging to the African (AFR) and Euroasian (EA) ancestry group (PC1 cutoff = .048; PC2 cutoff = .013; N = 102; Supplementary Fig. 4a) and one including only individuals within the European (EUR) ancestry group (PC1 cutoff = 0; PC2 cutoff = -0.049; N = 88; Supplementary Fig. 4a). Finally, after matching samples based on imaging data availability, for each of these clusters as well as for the whole KCL cohort, we evaluated the association of the C80 stratified PRS (C80-PRS) as well as its complementary score (C80-PRS-complementary) with [18F]-FDOPA uptake in the striatum indexed by Ki in the same identical fashion as the one reported in the main text (see Methods for details).

We found C80-PRS positively associated with greater striatal dopamine synthesis capacity as measured by [18F]-FDOPA specific uptake in NC and in patients with SCZ in the whole striatum and striatal subdivisions ROIs derived from Mawlawi, Martinez [16] and as described in McCutcheon, Beck [17] analysed in both ancestry clusters as well as the whole KCL cohort (Supplementary Fig. 4b). Interestingly, no significant association was found with complementary C80-PRS.

Exploratory genetic risk association and biological characterization analyses of the other identified components

We explored the association of the 69 identified components with SCZ PRS using the same multiple linear regression methods as reported in the main text (see Methods). We then looked at their biological characterization through enrichment and over-representation analyses to identify enrichment for SCZ risk genes as well as alternative pathways of risk convergence (see Methods for details). We found six additional components out of 69 associated with SCZ PRS at the nominal significance level (two-tailed α=.05, uncorrected; Supplementary Fig. 6a). None showed significant effects of diagnosis in postmortem samples. Of these, only one component (C102) was also enriched for SCZ risk genes (empirical p <.05; Supplementary Fig. 1a). C102 also showed enrichment for MDD, ASD, and PTSD risk genes as well as for SCZ differentially expressed genes (DEGs) previously observed in the CN, DLPFC, and HP (all empirical p <.05; Supplementary Fig. 1a). The tissue loading matrix revealed the C102 component to be most active in the HP (Supplementary Figure 1a). Accordingly, cell specificity analysis showed enrichment for glutamatergic synapses of hippocampal CA pyramidal and dentate gyrus neurons, among others (Supplementary Fig. 6b). Consistently, KEGG pathway analysis for C102 showed enrichment for glutamatergic synapse as well as for nicotine and morphine addiction-related genes (Supplementary Fig. 6c). Finally, we computed PRSs stratified for genes within each SCZ-PRS-associated component and evaluated their association with striatal dopamine synthesis capacity as measured by [18F]-FDOPA specific uptake in our discovery NC and SCZ cohorts (see Methods). Notably, none of the six components were significantly associated with striatal dopamine synthesis (Supplementary Fig. 6d). Additionally, in order to disentangle the specificity of the effects of C80-PRS on brain activity, we investigated the association of the six stratified PRSs with BOLD signal on the fMRI discovery cohort as previously described (see Methods). We observed no significant association with either reward anticipation or reward consumption (results not shown), further supporting the specificity of the C80-PRS for reward-related brain activity.

fMRI task layout

LIBD cohort. The general layout of a trial in this version of the MID task is divided into three phases: cue (2000-3000 ms), target (up to 1250 ms in the ‘difficult condition’, while up to 1750 ms in the ‘easy’ condition), and outcome (450 ms). Immediately prior to scanning, participants were instructed on the task to be performed in the scanner, including being explicitly informed of the meaning of each cue. Cues indicated, via background color, whether the trial would be more likely difficult, more likely easy, i.e., how long the target would likely stay on the screen, and the reward magnitude, i.e., $2 or $0 (control trial). The target was the appearance of paper currency (or blank paper in the control condition) over a money safe. Participants were instructed to respond with a single button press with their right thumb when the target appeared, and if the target was hit, i.e., response was made while the target was on the screen, the target (money bills or blank paper) fell into the money safe (450 ms). On the contrary, a trial was considered an error if the participant: a) did not respond to the target, b) pressed more than once to the target, or c) pressed prior to target appearance. In these cases, the target (money bills or blank paper) disappeared without falling into the money safe and participants did not receive money towards final payment. The outcome phase provided feedback for money earned from that trial. Intertrial intervals (2500-4500 s) were calculated to maintain the same number of trials regardless of reaction time variability. An extensive description of the task is provided by Kholi et al. 2018[18].

UNIBA cohort. The general layout of a trial in this version of the MID task is divided into three phases: cue (2000 ms), target (up to 500 ms), and outcome (2000 ms). Immediately prior to scanning, participants were instructed on the task to be performed in the scanner, including being explicitly informed of the meaning of each cue. The target presentation duration was calculated based on individual performance during the training phase to achieve at least 67% success rate. Cue stimuli consisted of three different white geometric shapes: a full circle, representing a chance of gain 100 points (reward condition), an empty circle, representing a chance of gain 0 point (control condition) and a full square, representing a chance of losing 100 points (punishment condition). The target was the appearance of a white triangle. Immediately after the response, feedback appeared for 2000 ms

documenting whether the participant had won or lost points as well as their cumulative total at that point. Participants were instructed to respond with a single button press with their right thumb when the target appeared to gain or to not lose points. A trial was considered a hit, when the response was made while the target was on the screen. On the contrary, a trial was considered an error if the participant: a) did not respond to the target, b) pressed prior to target appearance. The outcome phase provided feedback for money earned from that trial and the total amount earned so far.

Striatal parcellation of C80-related fMRI activations

To quantitatively assess the striatal subregions' overlap, we evaluated the distribution of the significant striatal clusters observed in the discovery and replication fMRI cohorts using canonical functional striatum ROI according to the parcellation described by Mawlawi, Martinez [16], which defined three subregions—associative, limbic, and sensorimotor (Supplementary Fig, 7a)—that underlie distinct functions of the striatum based on the cortical afferents each of these subregions projects or receives. We calculated the percentage of clusters where we independently found a significant TFCE-FDR<.05 effect of the C80-PRS, overlapping with the three striatal subregions, excluding voxels outside the grey matter. Both clusters predominantly fell within the associative striatum (Discovery=95%; replication=99%) with a minimal percentage in the limbic striatum in the discovery cohort (5%) and sensorimotor striatum in the replication cohort (1%). These findings suggests that the effect is convincingly related to activity in the same striatal sub-region encompassing the caudate nucleus (Supplementary Fig. 7b).

Furthermore, to quantify the extent of voxelwise overlap between the two independently detected clusters from discovery and replication fMRI analyses, we counted the numbers of spatially overlapping voxels corresponding to 6 (162 mm^3; Supplementary Fig. 7c). Notably, the overlap is located in the head of the CN, the same region used in the *postmortem* study. While the spatial correlation of fMRI signals may blur the accuracy of signal localization across different scanners and

experiments, the rest of these two overlapping clusters is still contained in the associative striatum as defined by Mawlawi, Martinez [16].

ROIs analysis on individual striatal fMRI activations

To account for the potential influence of individual variability in the localization of the C80-PRS effects on BOLD signal, we complemented the voxelwise analysis by extracting the individual signal from striatum ROIs using an uncorrected threshold of $\alpha<.005$[19]. We employed the six striatum ROIs (right and left associative, sensorimotor, and limbic subregions per Mawlawi, Martinez [16] and as described above). Subsequently, we performed associations of the C80-PRS with the signal extracted from the individual activation maps from the left and the right ROIs. Given that the uncorrected signal derived from the ROIs may be susceptible to type-I errors, post-hoc associations were adjusted for multiple comparisons to account for the number of tests conducted (k=6). Extracting the averaged signal from the associative striatum ROIs, we confirmed the significant positive associations on the signal extracted from the right associative striatum ROI (discovery: $t(84)=3.34$; $p_{[FDR]}=.006$; partial $R^2=0.12$; replication: $t(53)=3.45$; $p_{[FDR]}=.005$; partial $R^2=0.18$) along with significant positive associations on the signal extracted from the left associative striatum ROI, though with smaller effect sizes (discovery: $t(84)=2.39$; $p_{[FDR]}=.05$; partial $R^2=0.06$; replication: $t(53)=3.28$; $p_{[FDR]}=0.009$; partial $R^2=0.16$; Supplementary Fig. 8). No associations were significant in the limbic and sensorimotor striatum ($p_{[FDR]}>.05$) in either sample. Nevertheless, the potential influence of individual variability in signal localization, coupled with the limited sample sizes could have prevented the identification of under-threshold BOLD effects at the voxel-wise level on the left hemisphere.

Supplementary Figures

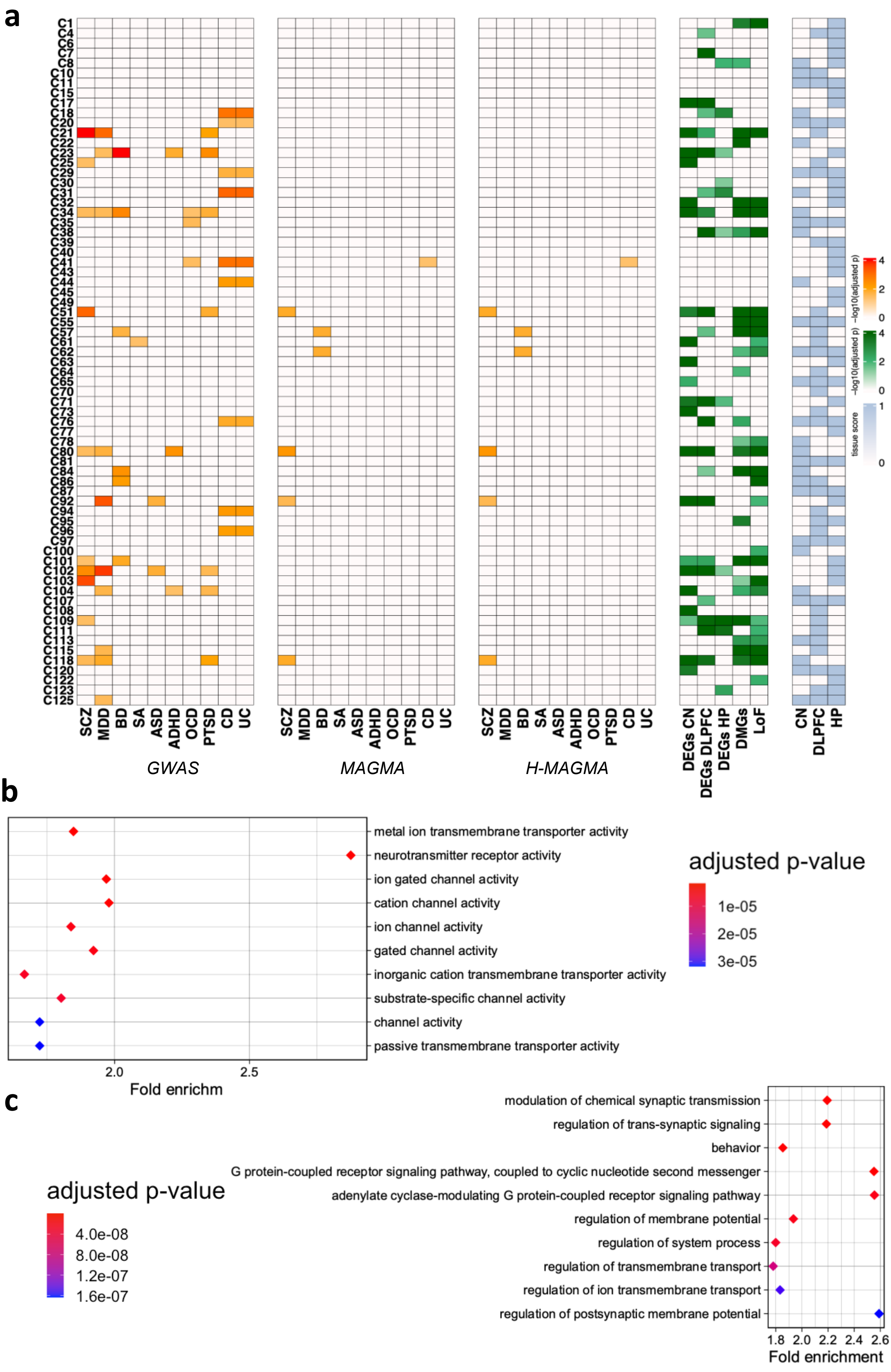
a
C1
C4
C6
C7
C8
C10
C11
C15
C17
C18
C20
C21
C22
C23
C25
C29
C30
C31
C32
C34
C35
C38
C39
C40
C41
C43
C44
C45
C49
C51
C55
C57
C61
C62
C63
C64
C65
C70
C71
C73
C76
C77
C78
C80
C81
C84
C86
C87
C92
C94
C95
C96
C97
C100
C101
C102
C103
C104
C107
C108
C109
C111
C113
C115
C118
C120
C122
C123
C125
SCZ MDD BD SA ASD ADHD OCD PTSD CD UC
GWAS
SCZ MDD BD SA ASD ADHD OCD PTSD CD UC
MAGMA
SCZ MDD BD SA ASD ADHD OCD PTSD CD UC
H-MAGMA
DEGs CN
DEGs DLPFC
DEGs HP
DMGs
LoF
CN
DLPFC
HP
−log10(adjusted p)
4
2
0
−log10(adjusted p)
4
2
0
tissue score
1
0
b
metal ion transmembrane transporter activity
neurotransmitter receptor activity
ion gated channel activity
cation channel activity
ion channel activity
gated channel activity
inorganic cation transmembrane transporter activity
substrate-specific channel activity
channel activity
passive transmembrane transporter activity
2.0
2.5
Fold enrichm
adjusted p-value
1e-05
2e-05
3e-05
c
modulation of chemical synaptic transmission
regulation of trans-synaptic signaling
behavior
G protein-coupled receptor signaling pathway, coupled to cyclic nucleotide second messenger
adenylate cyclase-modulating G protein-coupled receptor signaling pathway
regulation of membrane potential
regulation of system process
regulation of transmembrane transport
regulation of ion transmembrane transport
regulation of postsynaptic membrane potential
1.8 2.0 2.2 2.4 2.6
Fold enrichment
adjusted p-value
4.0e-08
8.0e-08
1.2e-07
1.6e-07

Supplementary Figure 1: 69 SDA components characterization analyses results and discovery C80 enriched gene ontologies.

a, Gene enrichment analysis results are shown for all 69 robust components. From the left, the first (GWAS), second (MAGMA) and third orange grids (H-MAGMA) show enrichment results for schizophrenia risk genes, other psychiatric illness risk genes, and immune condition risk genes. Enrichment testing results are shown for differentially expressed genes, differentially methylated genes, and loss of function variant intolerant genes in the green grid. The final lightblue grid show tissue specificity as determined by the tissue scores generated during the SDA process and reflects the relative contribution of component gene networks within each of the sampled regions to the overall component. Adjusted p-values shown are empirical p-values obtained from permutation tests (overrepresentation analysis: one-sided Fisher exact test).
See Fig. 2 caption for abbreviations.
b, and **c,** Gene ontology enrichment of C80 for biological processes and molecular function. Overrepresentation analysis was performed using the *clusterProfiler R*[20] package and FDR-adjusted p-values are reported. Diamonds represent fold enrichment (x-axis) for each Gene ontology category (y-axis) and are colored based on the respective adjusted p-value.

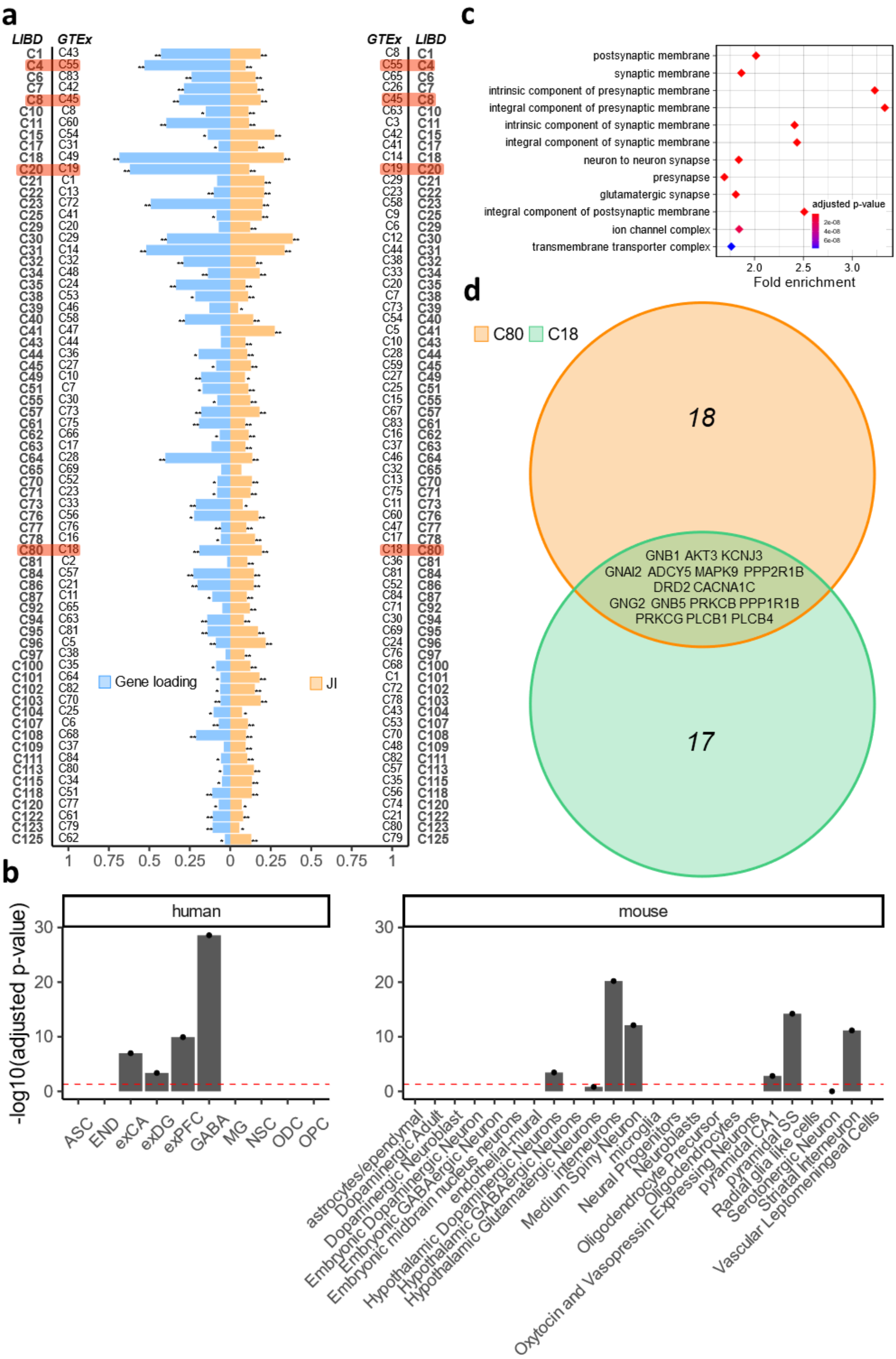

a
LIBD
GTEx
C1 C4 C6 C7 C8 C10 C11 C15 C17 C18 C20 C21 C22 C23 C25 C29 C30 C31 C32 C34 C35 C38 C39 C40 C41 C43 C44 C45 C49 C51 C55 C57 C61 C62 C63 C64 C65 C70 C71 C73 C76 C77 C78 C80 C81 C84 C86 C87 C92 C94 C95 C96 C97 C100 C101 C102 C103 C104 C107 C108 C109 C111 C113 C115 C118 C120 C122 C123 C125
C43 C55 C83 C42 C45 C8 C60 C54 C31 C49 C19 C1 C13 C72 C41 C20 C29 C14 C32 C48 C24 C53 C46 C58 C47 C44 C36 C27 C10 C7 C30 C73 C75 C66 C17 C28 C69 C52 C23 C33 C56 C76 C16 C18 C2 C57 C21 C11 C65 C63 C81 C5 C38 C35 C64 C82 C70 C25 C6 C68 C37 C84 C80 C34 C51 C77 C61 C79 C62
GTEx
LIBD
C8 C55 C65 C26 C45 C63 C3 C42 C41 C14 C19 C29 C23 C58 C9 C6 C12 C44 C38 C33 C20 C7 C73 C54 C5 C10 C28 C59 C27 C25 C15 C67 C83 C16 C37 C46 C32 C13 C75 C11 C60 C47 C17 C18 C36 C81 C52 C84 C71 C30 C69 C24 C76 C68 C1 C72 C78 C43 C53 C70 C48 C82 C57 C35 C56 C74 C21 C80 C79
C1 C4 C6 C7 C8 C10 C11 C15 C17 C18 C20 C21 C22 C23 C25 C29 C30 C31 C32 C34 C35 C38 C39 C40 C41 C43 C44 C45 C49 C51 C55 C57 C61 C62 C63 C64 C65 C70 C71 C73 C76 C77 C78 C80 C81 C84 C86 C87 C92 C94 C95 C96 C97 C100 C101 C102 C103 C104 C107 C108 C109 C111 C113 C115 C118 C120 C122 C123 C125
Gene loading
JI
1 0.75 0.5 0.25 0 0.25 0.5 0.75 1
b
-log10(adjusted p-value)
human
mouse
0 10 20 30
ASC END exCA exDG exPFC GABA MG NSC ODC OPC
astrocytes/ependymal
Dopaminergic Adult
Embryonic Dopaminergic Neuroblast
Embryonic Dopaminergic Neuron
Embryonic GABAergic Neuron
Embryonic midbrain nucleus neurons
endothelial-mural
Hypothalamic Dopaminergic Neurons
Hypothalamic GABAergic Neurons
Hypothalamic Glutamatergic Neurons
interneurons
Medium Spiny Neuron
microglia
Neural Progenitors
Neuroblasts
Oligodendrocyte Precursor
Oligodendrocytes
Oxytocin and Vasopressin Expressing Neurons
pyramidal CA1
pyramidal SS
Radial glia like cells
Serotonergic Neuron
Striatal Interneuron
Vascular Leptomeningeal Cells
c
postsynaptic membrane
synaptic membrane
intrinsic component of presynaptic membrane
integral component of presynaptic membrane
intrinsic component of synaptic membrane
integral component of synaptic membrane
neuron to neuron synapse
presynapse
glutamatergic synapse
integral component of postsynaptic membrane
ion channel complex
transmembrane transporter complex
adjusted p-value
2e-08
4e-08
6e-08
2.0 2.5 3.0
Fold enrichment
d
C80 C18
18
GNB1 AKT3 KCNJ3
GNAI2 ADCY5 MAPK9 PPP2R1B
DRD2 CACNA1C
GNG2 GNB5 PRKCB PPP1R1B
PRKCG PLCB1 PLCB4
17

Supplementary Figure 2: GTEx replication analyses results.

a, 69 SDA components generated from the LIBD discovery dataset replicated in the GTEx dataset using JI (orange bars) or gene loading correlation (light blue bars). (*) indicates statistical significance after one-sided permutations test < .05; (**) indicates statistical significance after one-sided permutations test < .001. JI values on the right and gene loading R^2 on the left x axis are shown. Discovery C80 and replication C18 are one of the 4 pairs of components highlighted in red that are consistent with both replication measures. Source data are provided as a Source Data file.
b, Cell-type specificity of replication component C18 using human (left) and mouse (right) single-cell atlases. Mean-rank Gene Set Test in the *limma R* package[21] was used to obtain enrichment p-values shown. y-axes show FDR-adjusted p-values after correcting for multiple comparisons across components (N = 69) and cell types (human atlas = 10; mouse atlas = 24). Red dashed lines represent $\alpha_{[FDR]}$=.05. Individual data points are shown using overlaid dot plots. Barplots demonstrates a higher specicifty for GABAergic, medium spiny and dopaminergic neurons. See Figure 2 caption for abbreviations. Source data are provided as a Source Data file.
c, Gene ontology enrichment of replication component C18 for cellular compartments. Overrepresentation analysis was performed using the *clusterProfiler R*[20] package and FDR-adjusted p-values are reported. Diamonds represent fold enrichment (x-axis) for each Gene ontology category (y-axis) and are colored based on the respective adjusted p-value.
d, Venn diagram showing intersection between C80 (orange) and C18 (green) genes enriched for KEGG term dopaminergic synapse with $p_{[FDR]}$<.05.

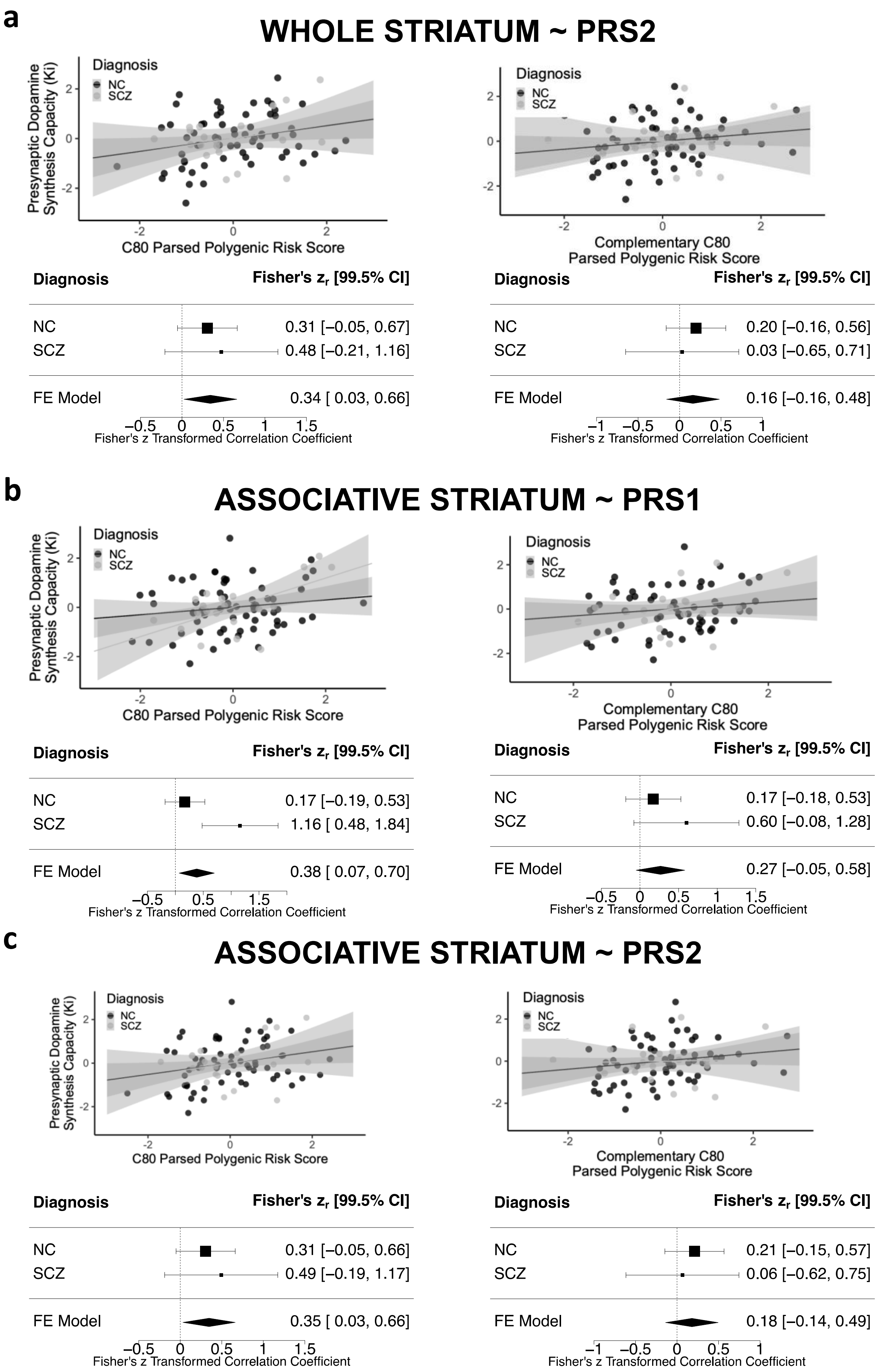
a
WHOLE STRIATUM ~ PRS2
Presynaptic Dopamine Synthesis Capacity (Ki)
Diagnosis
NC
SCZ
C80 Parsed Polygenic Risk Score
Complementary C80 Parsed Polygenic Risk Score
Diagnosis | Fisher's z_r [99.5% CI]
NC 0.31 [−0.05, 0.67]
SCZ 0.48 [−0.21, 1.16]
FE Model 0.34 [0.03, 0.66]
Fisher's z Transformed Correlation Coefficient
NC 0.20 [−0.16, 0.56]
SCZ 0.03 [−0.65, 0.71]
FE Model 0.16 [−0.16, 0.48]
b
ASSOCIATIVE STRIATUM ~ PRS1
NC 0.17 [−0.19, 0.53]
SCZ 1.16 [0.48, 1.84]
FE Model 0.38 [0.07, 0.70]
NC 0.17 [−0.18, 0.53]
SCZ 0.60 [−0.08, 1.28]
FE Model 0.27 [−0.05, 0.58]
c
ASSOCIATIVE STRIATUM ~ PRS2
NC 0.31 [−0.05, 0.66]
SCZ 0.49 [−0.19, 1.17]
FE Model 0.35 [0.03, 0.66]
NC 0.21 [−0.15, 0.57]
SCZ 0.06 [−0.62, 0.75]
FE Model 0.18 [−0.14, 0.49]

Supplementary Figure 3: C80 and complementary C80-PRS association with dopamine synthesis capacity in different striatal subdivisions.

a, Association between C80-PRS and complementary C80-PRS with whole-striatum dopamine synthesis capacity in the PET discovery cohort (*n* = 84 individuals; 64 NC and 20 SCZ). Scatter plots on the top show standardized individual mean K_i values for whole-striatum region of interest (ROI) plotted against C80-PRS2 (left) and complementary C80-PRS2 (right) for the neurotypical control and SCZ subjects. The forest plots of the respective metanalyses are shown on the right and left bottom. Mean fitted values and related shaded 95% confidence interval are shown in the scatterplots. Fisher's r-to-z transformed correlation coefficients and related 99.5% confidence interval are shown in the forest plot. Source data are provided as a Source Data file.

b, and **c,** Association between C80-PRS and complementary C80-PRS with associative-striatum dopamine synthesis capacity in the PET discovery cohort (*n* = 84 individuals; 64 NC and 20 SCZ). Scatter plots on the top show standardized individual mean K_i values for associative-striatum ROI plotted against C80-PRS1 and complementary C80-PRS1 (**b** left and right respectively) as well as C80-PRS2 and complementary C80-PRS2 (**c** left and right respectively) for the neurotypical control and SCZ subjects. The forest plots of the respective metanalyses are shown on the right and left bottom. Mean fitted values and related shaded 95% confidence interval are shown in the scatterplots. Fisher's r-to-z transformed correlation coefficients and related 99.5% confidence interval are shown in the forest plot. Source data are provided as a Source Data file.

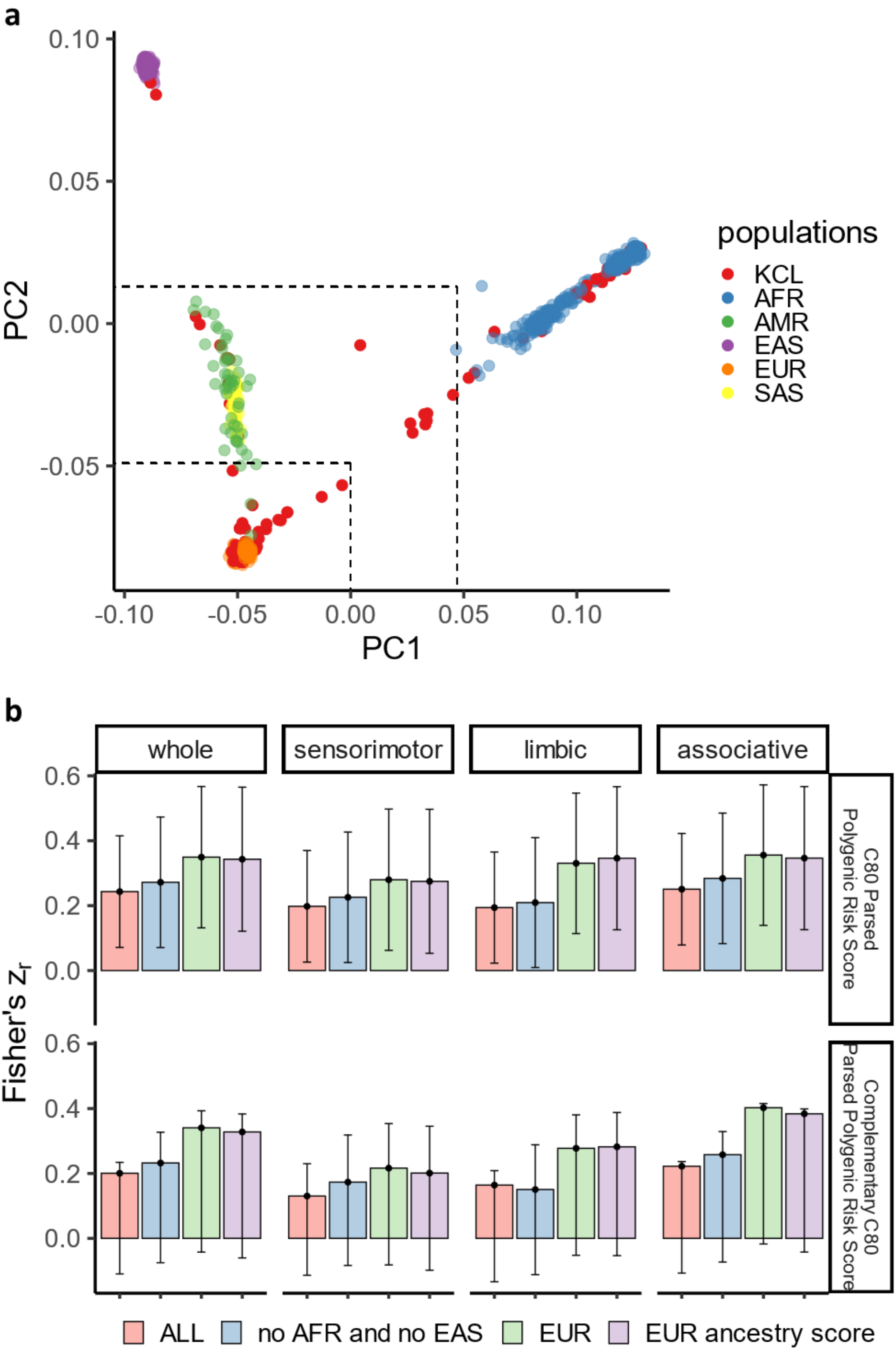
a
0.10
0.05
0.00
-0.05
PC2
-0.10
-0.05
0.00
0.05
0.10
PC1
populations
KCL
AFR
AMR
EAS
EUR
SAS
b
whole
sensorimotor
limbic
associative
0.6
0.4
0.2
0.0
Fisher's z_r
C80 Parsed Polygenic Risk Score
Complementary C80 Parsed Polygenic Risk Score
ALL
no AFR and no EAS
EUR
EUR ancestry score

Supplementary Figure 4: C80-PRS and complementary C80-PRS association with striatal dopamine synthesis capacity across different ancestry definitions.

a, Population stratification plot for discovery KCL cohort. The graph shows the overlap of the first two principal components based on genetic markers from KCL sample and a reference dataset (HapMap3; 5 super populations used). Red dots represent individuals in the KCL cohort. Black dashed lines delineate PC1 and PC2 cutoffs used to define the different ancestry clusters. Source data are provided as a Source Data file.

b, Associations between C80-PRS2 and complementary C80-PRS2 in multiple striatum subdivisions and across different ancestry clusters in the discovery KCL cohort are shown. Bar plots indicate meta-analytic Fisher's r-to-z transformed correlation coefficients obtained from respective meta-analyses performed for each striatal subdivision and each ancestry group. Error bars related to 95% confidence interval are also plotted. *ALL* indicates whole KCL cohort used; *no AFR and no EAS* indicates KCL samples excluding those overlapping with reference AFR and EAS ancestry; *EUR* indicates only KCL samples overlapping with reference EUR ancestry; *EUR ancestry score* indicates KCL samples included after ancestry score computation (see Methods for details). Source data are provided as a Source Data file.

Abbreviations: AFR: African; AMR: Ad Mixed American; EAS: East Asian; EUR: European; SAS: South Asian

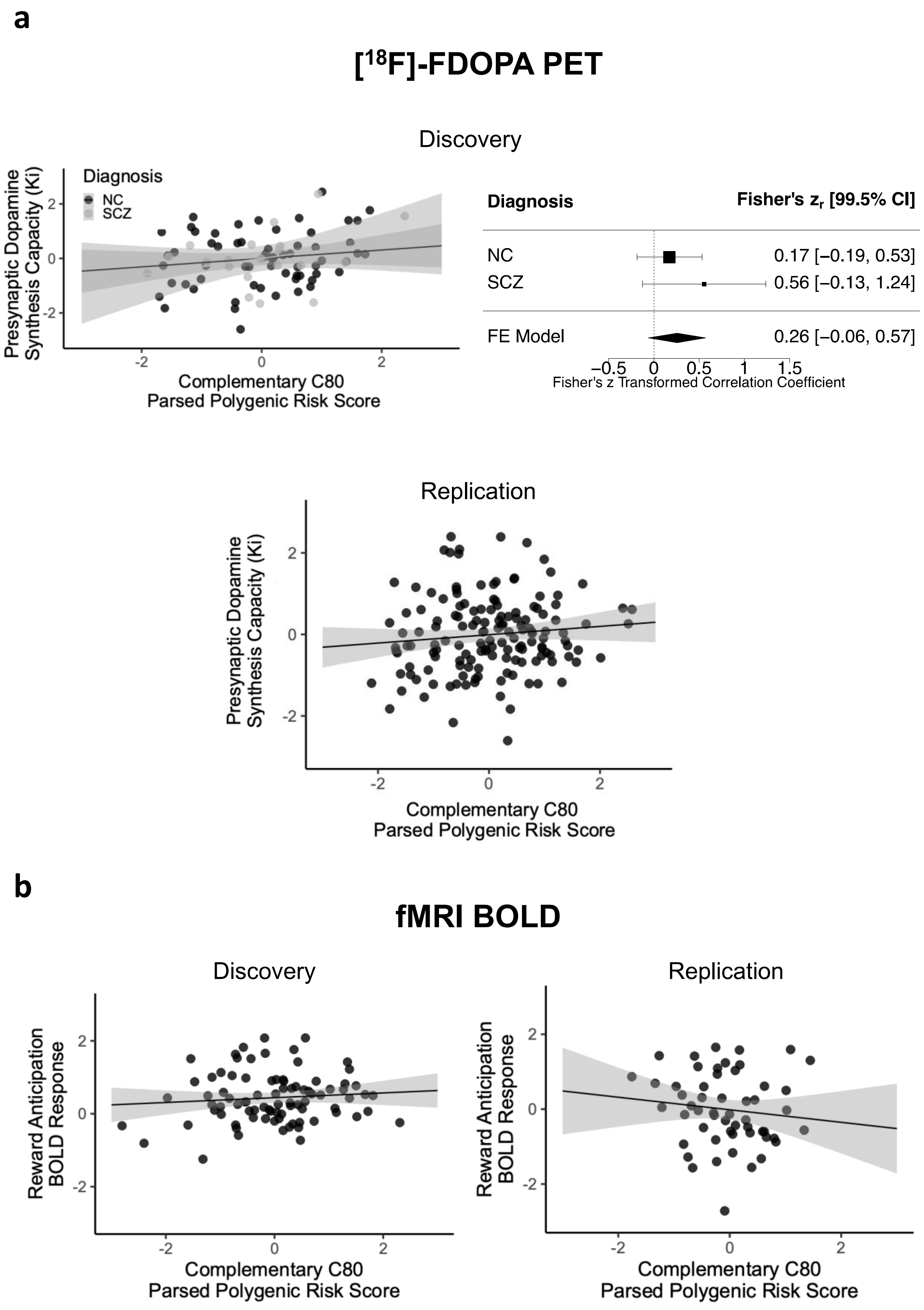
a
[18F]-FDOPA PET
Discovery
Presynaptic Dopamine Synthesis Capacity (Ki)
Diagnosis
NC
SCZ
Complementary C80 Parsed Polygenic Risk Score
Diagnosis
Fisher's z_r [99.5% CI]
NC
0.17 [−0.19, 0.53]
SCZ
0.56 [−0.13, 1.24]
FE Model
0.26 [−0.06, 0.57]
Fisher's z Transformed Correlation Coefficient
Replication
Presynaptic Dopamine Synthesis Capacity (Ki)
Complementary C80 Parsed Polygenic Risk Score
b
fMRI BOLD
Discovery
Replication
Reward Anticipation BOLD Response
Complementary C80 Parsed Polygenic Risk Score

Supplementary Figure 5: Complementary C80-PRS association with neuroimaging parameters: striatal dopamine synthesis capacity ([18F]-FDOPA PET) and reward anticipation-related fMRI activation (fMRI BOLD).

a, Associations between complementary C80-PRS and both PET cohorts are shown. First row (PET discovery; *n* = 84 individuals; 64 NC and 20 SCZ): scatter plot on the left shows individual mean K_i values for the whole-striatum region of interest (ROI) plotted against complementary C80-PRS for the neurotypical control and SCZ subjects while on the right the relative forest plot of the metanalysis is shown.

Second row (PET replication; *n* = 150 individuals): scatter plot shows individual mean K_i values for the whole-striatum ROI plotted against complementary C80-PRS.

Mean fitted values and related shaded 95% confidence interval are shown in the scatterplots. Fisher's r-to-z transformed correlation coefficients and related 99.5% confidence interval are shown in the forest plot. Source data are provided as a Source Data file. Source data are provided as a Source Data file.

b, Associations between complementary C80-PRS and both fMRI cohorts are shown. Scatter plots show standardized individual MID-related fMRI BOLD contrasts (discovery on the left: *n* = 86 neurotypical individuals; replication on the right: *n* = 55 neurotypical individuals) plotted against complementary C80-PRS. Mean fitted values and related shaded 95% confidence interval are shown in the scatterplots. Source data are provided as a Source Data file.

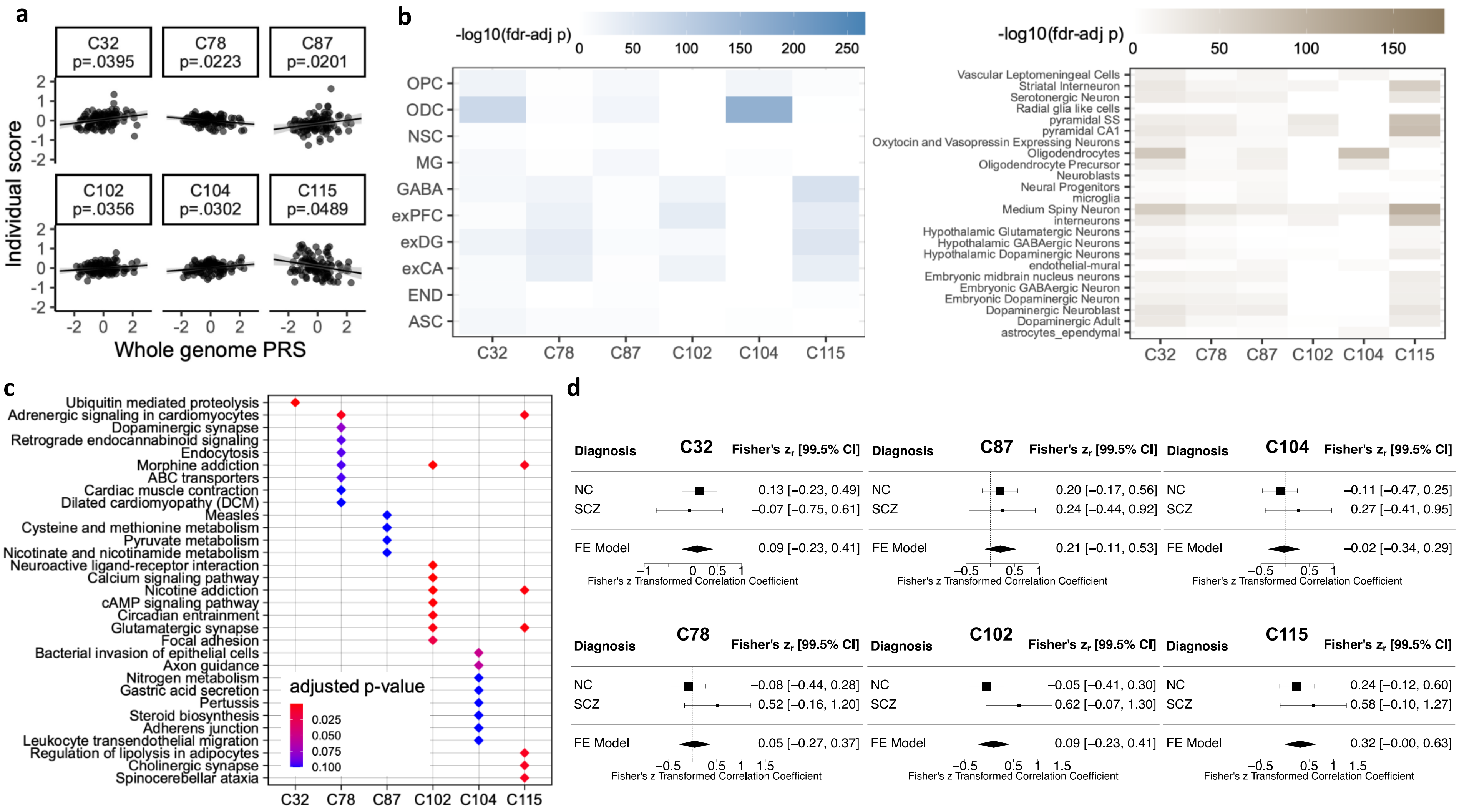

Supplementary Figure 6: Biological characterization, genetic risk, and striatal dopamine synthesis capacity association of other SDA components.

a, Scatter plots show SDA component scores (y-axis) as a function of polygenic risk for schizophrenia (x-axis) and include regression fit line with mean fitted values and related shaded 95% confidence interval. Nominal two-tailed p-values are shown. Source data are provided as a Source Data file.

b, Heatmaps show cell-type marker genes overrepresentation using human (light-blue) and mouse (brown) single-cell atlases. Mean-rank Gene Set Test in the *limma R* package[21] was used to obtain enrichment p-values shown. FDR-adjusted p-values after correcting for multiple comparisons across components (N = 69) and cell types (human atlas = 10; mouse atlas = 24) are shown. See Figure 2 caption for abbreviations.

c, KEGG enrichment of all six PRS-associated components. Overrepresentation analysis was performed using the *clusterProfiler R*[20] package and FDR-adjusted p-values are reported. Diamonds represent each KEGG category (y-axis) enriched for each component (x-axis) and are colored based on the respective adjusted p-value. **d**, Forest plots show metanalyses of the association between the six stratified PRSs with whole-striatum dopamine synthesis capacity in the PET discovery cohort. Fisher's r-to-z transformed correlation coefficients and related 99.5% confidence intervals are shown. Source data are provided as a Source Data file.

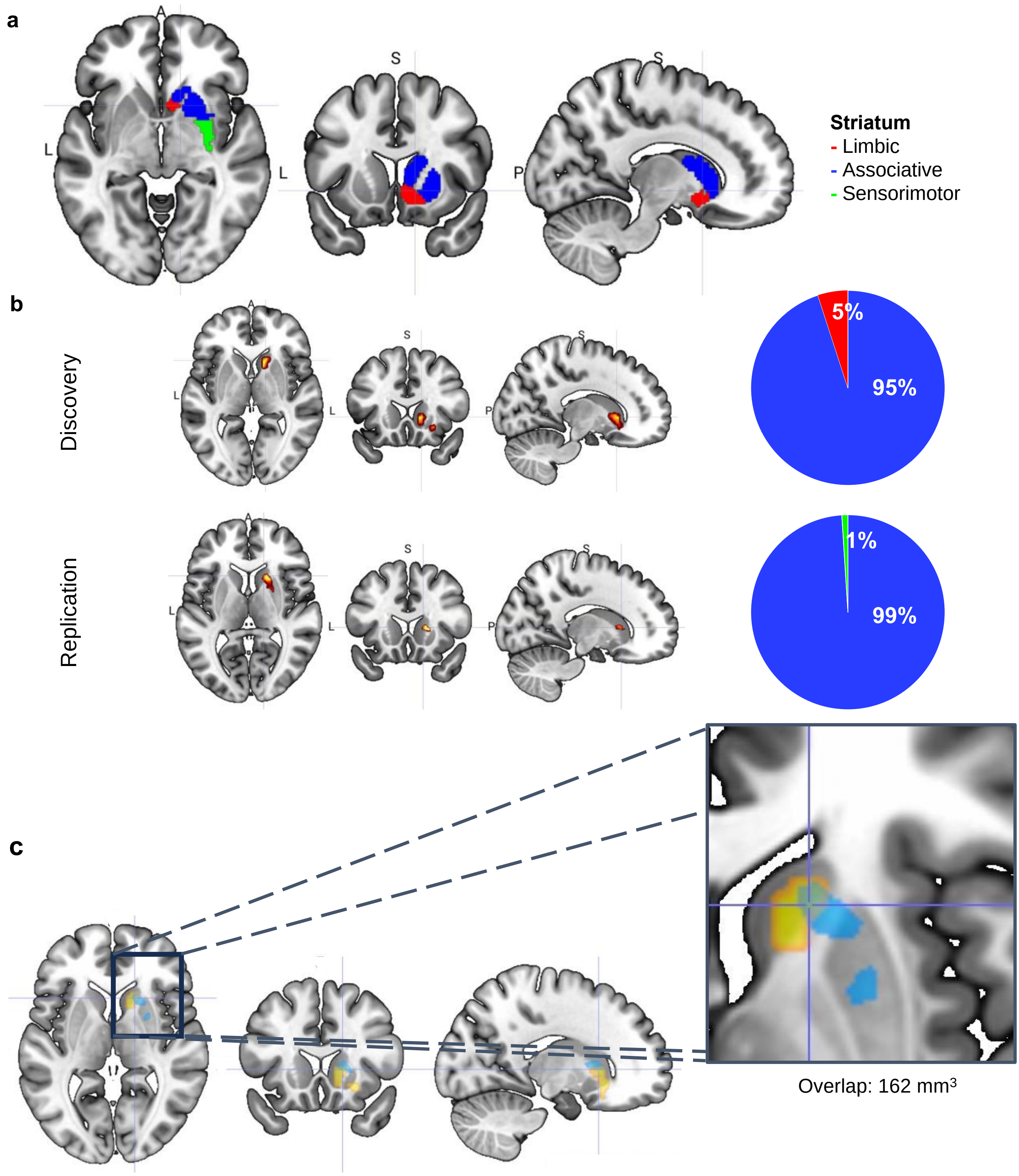

Supplementary Figure 7. Striatal parcellation of C80-PRS related BOLD activations during reward anticipation.

a, sections showing the localization of the right striatum ROIs divided in associative, limbic, and sensorimotor sub-regions depicted in blue, red, and green respectively.
b, Pie charts depicting the percentage of fMRI voxels associated with the C80 Parsed Polygenic Risk Score at the voxel-wise level in the discovery and replication samples (TFCE-FDR<.05) within each of the striatal sub-divisions.

c, Zooming section showing the overlap extension of the clusters significantly associated with the C80 Parsed Polygenic Risk Score at the voxel-wise level in the discovery (yellow) and replication (blue) samples (TFCE-FDR<.05) covering 6 voxels (162 mm3) within the right associative striatum ROI.

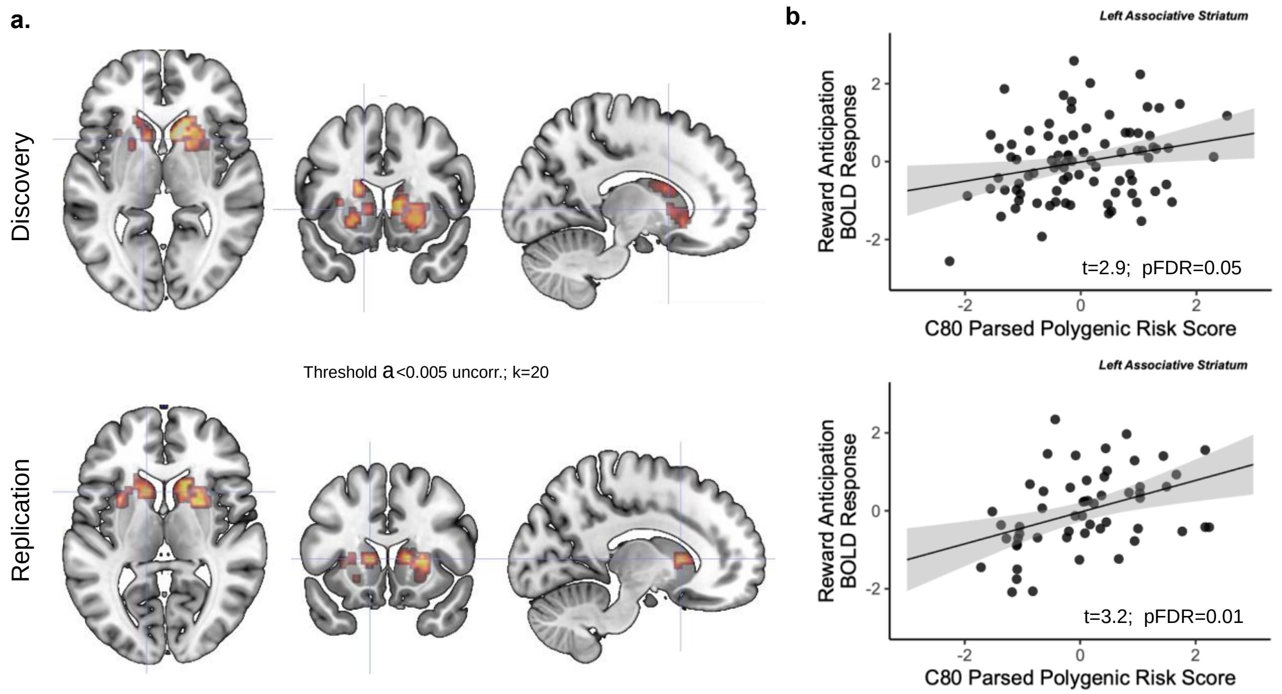

Supplementary Figure 8. ROIs analysis on individual striatal fMRI activations.

a, Sections showing the activation patterns at the group level within the bilateral striatum ROIs [16, 17] at α<.005, k=20 in the discovery (top; *n* = 86 neurotypical individuals) and replication (bottom; *n* = 55 neurotypical individuals) samples.

b, Scatterplot showing the associations between the signal extracted from the individual activation maps from the left associative striatum ROI and the C80-PRS in the discovery (top; *n* = 86 neurotypical individuals) and replication (bottom; *n* = 55 neurotypical individuals) samples. Mean fitted values and related shaded 95% confidence interval are shown in the scatterplots. Source data are provided as a Source Data file.

Supplementary Tables

Supplementary Table 1.

Demographics of cohorts used for neuroimaging association and ancestry stratification analyses are tabulated. Table is separated for samples whose imaging or genetic data is available.
Abbreviations: NC: Neurotypical controls; SCZ: Patients with schizophrenia; EUR: European

Modality	Cohort	Diagnosis (NC/SCZ)	N	Self-declared Ancestry
**Imaging**				
**[^{18}F]-FDOPA PET**	**Discovery**	NC	92	White British = 64<br>Black British = 19<br>Asian British = 4<br>Mixed = 5
		SCZ	47	/
	**Replication**	NC	150	EIUR: 150
**Reward fMRI**	**Discovery**	NC	86	EUR: 86
	**Replication**	NC	55	EUR: 55
**Genetic**				
**[^{18}F]-FDOPA PET**	**Discovery**	NC	121	
		SCZ	47	
	**Replication**	NC	169	
**Reward fMRI**	**Discovery**	NC	86	
	**Replication**	NC<br>SCZ	2,178	

Supplementary References

1. Purcell S*, et al.* PLINK: a tool set for whole-genome association and population-based linkage analyses. *Am J Hum Genet* **81**, 559-575 (2007).

2. Schizophrenia Working Group of the Psychiatric Genomics Consortium, Ripke S, Walters JT, O'Donovan MC. Mapping genomic loci prioritises genes and implicates synaptic biology in schizophrenia. *medRxiv*, (2020).

3. Lange K. *Mathematical and statistical methods for genetic analysis*. Springer (1997).

4. Ellingson SR, Fardo DW. Automated quality control for genome wide association studies. *F1000Res* **5**, 1889 (2016).

5. Wright S. Coefficients of Inbreeding and Relationship. *The American Naturalist* **56**, 330-338 (1922).

6. Gross A, Tönjes A, Scholz M. On the impact of relatedness on SNP association analysis. *BMC Genet* **18**, 104 (2017).

7. Howie B, Marchini J, Stephens M. Genotype imputation with thousands of genomes. *G3 (Bethesda)* **1**, 457-470 (2011).

8. Delaneau O, Marchini J, Zagury JF. A linear complexity phasing method for thousands of genomes. *Nat Methods* **9**, 179-181 (2011).

9. Zheng X, Levine D, Shen J, Gogarten SM, Laurie C, Weir BS. A high-performance computing toolset for relatedness and principal component analysis of SNP data. *Bioinformatics* **28**, 3326-3328 (2012).

10. Freeman B, Smith N, Curtis C, Huckett L, Mill J, Craig IW. DNA from buccal swabs recruited by mail: evaluation of storage effects on long-term stability and suitability for multiplex polymerase chain reaction genotyping. *Behav Genet* **33**, 67-72 (2003).

11. Anderson CA, Pettersson FH, Clarke GM, Cardon LR, Morris AP, Zondervan KT. Data quality control in genetic case-control association studies. *Nat Protoc* **5**, 1564-1573 (2010).

12. Loh PR*, et al.* Reference-based phasing using the Haplotype Reference Consortium panel. *Nat Genet* **48**, 1443-1448 (2016).

13. McCarthy S*, et al.* A reference panel of 64,976 haplotypes for genotype imputation. *Nat Genet* **48**, 1279-1283 (2016).

14. Conomos MP, Miller MB, Thornton TA. Robust inference of population structure for ancestry prediction and correction of stratification in the presence of relatedness. *Genet Epidemiol* **39**, 276-293 (2015).

15. Gogarten SM*, et al.* Genetic association testing using the GENESIS R/Bioconductor package. *Bioinformatics* **35**, 5346-5348 (2019).

16. Mawlawi O*, et al.* Imaging human mesolimbic dopamine transmission with positron emission tomography: I. Accuracy and precision of D(2) receptor parameter measurements in ventral striatum. *Journal of cerebral blood flow and metabolism : official journal of the International Society of Cerebral Blood Flow and Metabolism* **21**, 1034-1057 (2001).

17. McCutcheon R, Beck K, Jauhar S, Howes OD. Defining the Locus of Dopaminergic Dysfunction in Schizophrenia: A Meta-analysis and Test of the Mesolimbic Hypothesis. *Schizophr Bull* **44**, 1301-1311 (2018).

18. Kohli A*, et al.* Using Expectancy Theory to quantitatively dissociate the neural representation of motivation from its influential factors in the human brain: An fMRI study. *NeuroImage* **178**, 552-561 (2018).

19. Sacchet MD, Knutson B. Spatial smoothing systematically biases the localization of reward-related brain activity. *Neuroimage* **66**, 270-277 (2013).

20. Yu G, Wang LG, Han Y, He QY. clusterProfiler: an R package for comparing biological themes among gene clusters. *OMICS* **16**, 284-287 (2012).

21. Wu D, Smyth GK. Camera: a competitive gene set test accounting for inter-gene correlation. *Nucleic Acids Research* **40**, e133-e133 (2012).
