# Supplementary material for: Dopamine signaling enriched striatal gene set predicts striatal dopamine synthesis and physiological activity in vivo
Source: Nat Commun. 2024 Apr 30;15:3342. doi: 10.1038/s41467-024-47456-5 (PMC11061310; doi:10.1038/s41467-024-47456-5)
Supplement: Supplementary file 3 — Description of Additional Supplementary Files [file 41467_2024_47456_MOESM3_ESM.pdf]

Description of Additional Supplementary Files:

Supplementary Data 1: Compressed tar-zipped directory containing output of SDA computed on the LIBD discovery and GTEx replication datasets: individual score, tissue score, gene loading and posterior inclusion probability (PIP) matrices. The gene.loading_PIP_0.5.txt matrix refers to the gene loading matrix in which loadings for genes with PIP < .5 has been set to 0. This was the matrix used to infer component membership for each gene.

Supplementary Data 2: Excel file with LIBD discovery components summary information, association with both biological covariates and technical confounders (Table S1), and component membership (Table S2).

Table S1: Association between all SDA components and both biological covariates and technical confounders (p-values are reported). The number of genes included in each component is listed, and the 69 components carried forward for further analysis are indicated (column name: removed; value = No).
Table S2: Information about genes assigned to the 69 SDA components. For each gene and for each component a value of 1 indicates that gene is assigned to that specific component.

Supplementary Data 3: Excel file of discovery C80 (Table S1) and replication C18 (Table S2) genes as wells as relative GO and KEGG enrichment results (Table S3 and S4).

Table S1 and Table S2: List of genes belonging to discovery C80 and replication C18 components. Ensembl IDs are relative to GENCODE release 25.

Table S3 and Table S4: GO and KEGG results are reported for terms with p[FDR]<.05

Supplementary Data 4: Compressed zipped directory containing deidentified PRSs and technical covariates for all discovery and replication cohorts along with aggregated deidentified PET and fM RIdata used.

Supplementary Software: R scripts used for the analyses conducted
