# Supplementary material for: Dopamine signaling enriched striatal gene set predicts striatal dopamine synthesis and physiological activity in vivo
Source: Nat Commun. 2024 Apr 30;15:3342. doi: 10.1038/s41467-024-47456-5 (PMC11061310; doi:10.1038/s41467-024-47456-5)
Supplement: Supplementary file 9 — Reporting Summary [file 41467_2024_47456_MOESM9_ESM.pdf]

nature portfolio

Corresponding author(s): Giulio Pergola
Daniel R. Weinberger

Last updated by author(s): Mar 11, 2024

Reporting Summary

Nature Portfolio wishes to improve the reproducibility of the work that we publish. This form provides structure for consistency and transparency in reporting. For further information on Nature Portfolio policies, see our Editorial Policies and the Editorial Policy Checklist.

Statistics

For all statistical analyses, confirm that the following items are present in the figure legend, table legend, main text, or Methods section.

n/a	Confirmed	
☐	☒	The exact sample size (n) for each experimental group/condition, given as a discrete number and unit of measurement
☒	☐	A statement on whether measurements were taken from distinct samples or whether the same sample was measured repeatedly
☐	☒	The statistical test(s) used AND whether they are one- or two-sided<br>*Only common tests should be described solely by name; describe more complex techniques in the Methods section.*
☐	☒	A description of all covariates tested
☐	☒	A description of any assumptions or corrections, such as tests of normality and adjustment for multiple comparisons
☐	☒	A full description of the statistical parameters including central tendency (e.g. means) or other basic estimates (e.g. regression coefficient) AND variation (e.g. standard deviation) or associated estimates of uncertainty (e.g. confidence intervals)
☐	☒	For null hypothesis testing, the test statistic (e.g. F, t, r) with confidence intervals, effect sizes, degrees of freedom and P value noted<br>*Give P values as exact values whenever suitable.*
☐	☒	For Bayesian analysis, information on the choice of priors and Markov chain Monte Carlo settings
☒	☐	For hierarchical and complex designs, identification of the appropriate level for tests and full reporting of outcomes
☐	☒	Estimates of effect sizes (e.g. Cohen's d, Pearson's r), indicating how they were calculated

Our web collection on statistics for biologists contains articles on many of the points above.

Software and code

Policy information about availability of computer code

Data collection	No software was used for data collection
Data analysis	Codes used for the analyses presented in this paper are available at: https://zenodo.org/records/10699265<br>Software packages used in data analysis: SDA for tensor decomposition and gene co-expression analysis; R (version 3.6) for data processing, statistical analyses and data visualization; PLINK (v1.90b6.22) and PLINK (v2.00a3.6) for genotype data filtering and genomic eigenvariates computation; Michigan Imputation Server for genotype imputation; PRSice (2.3.3) for polygenic risk score computation; MAGMA (1.09b) for gene-set enrichment analysis; limma (3.46.0) for cell-type specificity analysis; clusterProfiler (3.18) for Gene Ontology enrichment analysis; MATLAB (2023a) and SPM12 for processing and statistical analysis of fMRI data; ANTs (2.5.0), PMOD (4.4) and SPM12 for processing and statistical analyses of PET data; MRIcro (1.9.1) for fMRI and PET data visualization.

For manuscripts utilizing custom algorithms or software that are central to the research but not yet described in published literature, software must be made available to editors and reviewers. We strongly encourage code deposition in a community repository (e.g. GitHub). See the Nature Portfolio guidelines for submitting code & software for further information.

Data

Policy information about availability of data

All manuscripts must include a data availability statement. This statement should provide the following information, where applicable:

- Accession codes, unique identifiers, or web links for publicly available datasets
- A description of any restrictions on data availability
- For clinical datasets or third party data, please ensure that the statement adheres to our policy

The LIBD post-mortem raw RNA-Seq FASTQ files for CN, DLPFC and HP are available through the database of Genotypes and Phenotypes (dbGap) and Globus collections (CN: phs003495.v1.p1 [https://www.ncbi.nlm.nih.gov/projects/gap/cgi-bin/study.cgi?study_id=phs003495.v1.p1]; DLPFC: jhpce#bsp2-dlpfc [http://research.libd.org/globus/jhpce_bsp2-dlpfc/index.html]; HP: jhpce#bsp2-hippo [http://research.libd.org/globus/jhpce_bsp2-hippo/index.html]).
The LIBD post-mortem raw genotype data are available through dbGap under accession code phs000979.v3.p2 [https://www.ncbi.nlm.nih.gov/projects/gap/cgi-bin/study.cgi?study_id=phs000979.v3.p2]).
The LIBD post-mortem processed RNA-seq data and accession codes to raw RNA-Seq FASTQ files and genotypes used in this study are also publicly available at: https://eqtl.brainseq.org/phase2/ and at: https://erwinpaquolalab.libd.org/caudate_eqtl/.
The GTEx post-mortem raw RNA-Seq FASTQ files for CN (GTEx tissue name: Brain - Caudate (basal ganglia)), DLPFC (GTEx tissue name: Brain - Frontal Cortex (BA9)) and HP (GTEx tissue name: Brain - Hippocampus) are available through dbGap with accession code phs000424.v8.p2 [https://www.science.org/doi/10.1126/science.1262110].
The GTEx post-mortem processed RNA-seq data used in this study are publicly available at: https://gtexportal.org/home/downloads/adult-gtex/bulk_tissue_expression.
The individual-level raw data for the discovery PET cohorts from KCL supporting the findings of this study are available from The NeurOimaging DatabasE (NODE) institutional repository (Institute of Psychiatry, Psychology & Neuroscience, King's College London) and can be accessed from co-author O.D.H. (oliver.howes@kcl.ac.uk) upon request. The authors will allow analysis of data with restricted access within one year from the request, according to extant regulations.
The individual-level raw data for the replication NIMH PET cohort, the LIBD fMRI discovery cohort, and the UNIBA fMRI replication cohort are not currently publicly available because the regulation at the time of consent acquisition required participants to explicitly consent to sharing data with select institutions and impedes data sharing unbeknown to participants.
The SCZ GWAS summary statistics used in this study are publicly available at:
https://figshare.com/articles/dataset/scz2022/19426775.
GRCh38 human genome reference genome is available here:
https://ftp.ebi.ac.uk/pub/databases/gencode/Gencode_human/release_25/GRCh38.p7.genome.fa.gz
The GENCODE release 25, GRCh38.p7 annotation file used in this study is available at:
https://ftp.ebi.ac.uk/pub/databases/gencode/Gencode_human/release_25/gencode.v25.basic.annotation.gtf.gz.
SDA output data are available in the Supplementary Data 1.
Components summary information is available in the Supplementary Data 2.
GO enrichment results are available in the Supplementary Data 3.
The aggregated deidentified PET and fMRI data along with PRSs and technical covariates for all discovery and replication cohorts used in this study are available in the Supplementary Data 4.
To ensure the replicability of the results reported in this work, all processed and aggregated data (Supplementary Data 1-4), SDA input data and SNPs used to compute C80-PRSs are also available at: https://doi.org/10.5281/zenodo.10699265.
Source data are provided with this paper.

Research involving human participants, their data, or biological material

Policy information about studies with human participants or human data. See also policy information about sex, gender (identity/presentation), and sexual orientation and race, ethnicity and racism.

Reporting on sex and gender

Demographic information on study participants are shown in Table 1. Sex was determined based on self-reporting and was used as confounding variable in all regression analyses.

Reporting on race, ethnicity, or other socially relevant groupings

Demographic information on study participants are shown in Table 1.
Ancestry was determined based on self-reporting for all samples.
For the association between SDA components and diagnosis, we included ancestry as covariate.
For the association between parsed PRS and PET and fMRI data, as summary statistics used are mainly based on the European population and PRS association with other race groups might lead to biases, we evaluated the individual ancestry based on the genotype data rather than only considering the self-reported race for all cohorts included for the brain function association analysis. Further details are provided in the Methods section.

Population characteristics

Demographic information on study participants are shown in Table 1 and Supplementary Table 1.
Postmortem data discovery LIBD - 238 samples with the following covariate-relevant population characteristics: diagnosis (154 neurotypical controls; 84 patients with schizophrenia), age (mean 46.6, std 16.8), sex (70 females; 168 males), ancestry (129 African ancestry; 109 European ancestry).
Postmortem data replication GTEx - 120 samples with the following covariate-relevant population characteristics: diagnosis (120 neurotypical controls), age (mean 59.2 std 9.6), sex (30 females; 90 males), ancestry (120 European ancestry).
[' F]-FDOPA PET discovery KCL - 85 samples with the following covariate-relevant population characteristics: diagnosis 65 neurotypical controls; 20 patients with schizophrenia), age (mean 29.3, std 8.2), sex (34 females; 51 males), ancestry (85

	European ancestry).<br>(1 F]-FDOPA PET replication NIH - 150 samples with the following covariate-relevant population characteristics: diagnosis (150 neurotypical controls), age (mean 35, std 11), sex (75 females; 75 males), ancestry (150 European ancestry).<br>Reward fMRI discovery LIBD - 86 samples with the following covariate-relevant population characteristics: diagnosis (86 neurotypical controls), age (mean 32, std 6), sex (47 females; 39 males), ancestry (86 European ancestry).<br>Reward fMRI replication BARI - 55 samples with the following covariate-relevant population characteristics: diagnosis (55 neurotypical controls), age (mean 26, std 6), sex (34 females; 21 males), ancestry (55 European ancestry).
Recruitment	Human postmortem brain tissues and imaging data were collected at several sites for this study. Partecipants recruitment is detailed in the Methods section.
Ethics oversight	The research described herein complies with all relevant ethical regulations. Postmortem human brain tissue was obtained as previously described. Briefly, tissues were primarily obtained by autopsy from the Offices of the Chief Medical Examiner of the District of Columbia and of the Commonwealth of Virginia, Northern District, all with informed consent from the legal next of kin (protocol 90-M-0142 approved by the National Institute of Mental Health (NIMH)/National Institutes of Health (NIH) Institutional Review Board). The National Institute of Child Health and Human Development Brain and Tissue Bank for Developmental Disorders (https://medschool.umaryland.edu/BTBank) provided infant, child and adolescent brain tissue samples under the NO1-HD-43368 and NO1-HD-4-3383 contracts. Additionally, donations of postmortem human brain tissue from patients with SCZ were provided with informed consent by next of kin from the Office of the Chief Medical Examiner for the State of Maryland under protocol number 12-24 from the State of Maryland Department of Health and Mental Hygiene and from the Office of the Medical Examiner, Department of Pathology, Homer Stryker, Maryland School of Medicine under protocol number 20111080 from the Western Institute Review Board. The Institutional Review Board of the University of Maryland at Baltimore and the State of Maryland approved the study protocol. The Lieber Institute for Brain Development (LIBD) received the tissues by donation under the terms of a material transfer agreement.<br>The discovery cohort of participants in the PET study was obtained under ethical permission given by the Administration of Radioactive Substances Advisory Committee (ARSAC), the South London and Maudsley/Institute of Psychiatry NHS Trust, the London Bentham Research Ethics Committee, and the Hammersmith Research Ethics Committee. All participants provided written, informed consent per King's College London (KCL) IRB-approved protocols.<br>The replication cohort of participants in the PET study was obtained under ethical permission given by the National Institute of Mental Health Institutional Review Board and National Institutes of Health (NIH) Radiation Safety Committee. All participants provided written, informed consent per NIH IRB-approved protocols.<br>The discovery cohort of participants in the fMRI experiments had no history of any psychiatric or neurological disorders and gave written, informed consent for a protocol approved by the NIH Combined Neurosciences IRB. Participants were told that they would be monetarily compensated based on earnings in the task.<br>The replication cohort of participants in the fMRI experiments had no history of any psychiatric or neurological disorders and gave informed consent for a protocol approved by the institutional ethics committee of from the University of Bari Aldo Moro (UNIBA). Participants were told that they would be compensated with one gift gadget (pen, t-shirt, pin, bag, pouch, notebook) when they earned at least 1700 points, and the chance of choosing between two or three gifts of their choice (when reaching 1900 and 2300 points, respectively) and encouraged to respond as quickly as possible.<br>The study design and conduct complied with all relevant regulations regarding the use of human study participants and was conducted in accordance with the criteria set by the Declaration of Helsinki.

Note that full information on the approval of the study protocol must also be provided in the manuscript.

Field-specific reporting

Please select the one below that is the best fit for your research. If you are not sure, read the appropriate sections before making your selection.

☒ Life sciences ☐ Behavioural & social sciences ☐ Ecological, evolutionary & environmental sciences

For a reference copy of the document with all sections, see nature.com/documents/nr-reporting-summary-flat.pdf

Life sciences study design

All studies must disclose on these points even when the disclosure is negative.

Sample size	Gene co-expression analysis: 238 samples for discovery cohort and 120 for replication cohort.<br>parsed PRS association with PET data: 85 samples for discovery cohort and 150 for replication cohort.<br>parsed PRS association with fMRI data: 86 samples for discovery cohort and 55 for replication cohort.<br>This is the first study employing tensor decomposition to analyze gene co-expression in the human brain. The sample size was determined by brain tissue available and funds available for sequencing. Previous studies (DOI: 10.1126/sciadv.ade2812; https://doi.org/10.1371/journal.pgen.1008549; https://doi.org/10.1038/tp.2016.253) performing gene co-expression analysis suggest this sample size is adequately powered to identify co-expressed gene sets using tensor decomposition. Previous studies (35871219; https://doi.org/10.1038/s41467-021-23694-9) associating genetic scores with neuroimaging data with similar sample sizes suggest the sample size used is well-powered for prediction of neuroimaging phenotype using parsed PRS.
Data exclusions	For gene co-expression analyses we removed samples with RNA integrity number < 0.6 and tissue-specific outliers deviating more than three standard deviations from the mean (see Methods for details) to obtain samples with high quality RNA expression data and to limit the impact of deviations from normality in expression data (CN=4; HP=7; DLPFC=5).<br>As the summary statistics used are mainly based on the European population and PRS association with other ancestry groups might lead to biases, we evaluated the individual ancestry based on the genotype data rather than only considering the self-reported ancestry for all

	cohorts included for the brain function association analysis (see Methods for details; KCL: 59; NIMH: 3; UNIBA: 213).
Replication	To replicate gene co-expression sets obtained with the LIBD data, we applied SDA on CN, HP, and DLPFC GTEx RNA-seq data using the exact same pipeline. Two replication measures were assessed: correlation between LIBD and GTEx component-specific gene loadings and Jaccard Index (JI) as the intersection/union of the LIBD and GTEx component-specific genes.<br>For the replication of the brain functional association analyses performed on the PET and fMRI discovery cohorts, we performed the same regression analysis using the parsed PRS as predictor and dopamine synthesis (PET ki) or striatal activation (BOLD signal) as dependent variables in two independent replication cohorts respectively.<br>We confirm all replication analyses were successfull as reported in the manuscript.
Randomization	This is an observational study from postmortem human brain tissue and thus subjects were not randomized into outcome groups.
Blinding	Investigators were not blinded to group allocation since the study is observational.

Reporting for specific materials, systems and methods

We require information from authors about some types of materials, experimental systems and methods used in many studies. Here, indicate whether each material, system or method listed is relevant to your study. If you are not sure if a list item applies to your research, read the appropriate section before selecting a response.

Materials & experimental systems

n/a	Involved in the study
☒	☐ Antibodies
☒	☐ Eukaryotic cell lines
☒	☐ Palaeontology and archaeology
☒	☐ Animals and other organisms
☒	☐ Clinical data
☒	☐ Dual use research of concern
☒	☐ Plants

Methods

n/a	Involved in the study
☒	☐ ChIP-seq
☒	☐ Flow cytometry
☐	☒ MRI-based neuroimaging

Plants

Seed stocks	*Report on the source of all seed stocks or other plant material used. If applicable, state the seed stock centre and catalogue number. If plant specimens were collected from the field, describe the collection location, date and sampling procedures.*
Novel plant genotypes	*Describe the methods by which all novel plant genotypes were produced. This includes those generated by transgenic approaches, gene editing, chemical/radiation-based mutagenesis and hybridization. For transgenic lines, describe the transformation method, the number of independent lines analyzed and the generation upon which experiments were performed. For gene-edited lines, describe the editor used, the endogenous sequence targeted for editing, the targeting guide RNA sequence (if applicable) and how the editor was applied.*
Authentication	*Describe any authentication procedures for each seed stock used or novel genotype generated. Describe any experiments used to assess the effect of a mutation and, where applicable, how potential secondary effects (e.g. second site T-DNA insertions, mosiacism, off-target gene editing) were examined.*

Magnetic resonance imaging

Experimental design

Design type	modified version of the MID task (see method section for details).
Design specifications	Details about the task layout are reported in the Supplementary materials.
Behavioral performance measures	Details about the variable recorded are reported in the method section.

Acquisition

Imaging type(s)	functional
Field strength	3T
Sequence & imaging parameters	Gradient-recall echo-planar imaging was used with the following parameters: TR = 2000 ms; TE=38 ms; flip angle=90; 64x64 matrix; FOV=240mm; and 38 3.6mm slices acquired with an interleaved order of slice acquisition.
Area of acquisition	Whole brain scan was used

Diffusion MRI ☐ Used ☒ Not used

Preprocessing

Preprocessing software	SPM12
Normalization	Mean functional-image driven spatial normalization to MNI space.
Normalization template	Details are reported in the method section.
Noise and artifact removal	35 3.5 mm slices acquired with an interleaved order of slice acquisition and first five frames discarded to allow steady-state magnetization. Slice timing correction, six-parameter coregistration to adjust for movement. At the model estimation stage, the data were high-pass filtered with a cutoff of 128 s to remove low-frequency drifts.
Volume censoring	spatial smoothing with an 8 mm Gaussian kernel were applied and yielded time series data with 3 mm isotropic resolution.

Statistical modeling & inference

Model type and settings	primary outcome measure was the contrast in BOLD signal of rewarded relative to control cue events. Details in the method section.
Effect(s) tested	Age, sex, IQ and first three GEs were used as covariates whereas MID-related BOLD signal (cue-related anticipatory response during reward versus control trials) was the dependent variable and PRS was the independent variable. Details in the method section.

Specify type of analysis: ☒ Whole brain ☐ ROI-based ☐ Both

Statistic type for inference (See Eklund et al. 2016)	voxel-wise
Correction	We considered the threshold free cluster enhancement correction p[TFCE-FDR] < 0.05 accounting for multiple comparisons. Details in the method section.

Models & analysis

n/a	Involved in the study
☒	☐ Functional and/or effective connectivity
☒	☐ Graph analysis
☒	☐ Multivariate modeling or predictive analysis
